# Yb_2_O_3_ Doped Zr_0.92_Y_0.08_O_2-α_(8YSZ) and Its Composite Electrolyte for Intermediate Temperature Solid Oxide Fuel Cells

**DOI:** 10.3390/ma11101824

**Published:** 2018-09-25

**Authors:** Yumin Cui, Ruijuan Shi, Junlong Liu, Hongtao Wang, Huiquan Li

**Affiliations:** Anhui Provincial Key Laboratory for Degradation and Monitoring of Pollution of the Environment, School of Chemical and Material Engineering, Fuyang Normal College, Fuyang 236037, China; cymlh@126.com (Y.C.); rjshi@fync.edu.cn (R.S.); jlliu@fync.edu.cn (J.L.)

**Keywords:** double doped ZrO_2_, composite, electrolyte, fuel cell, conductivity

## Abstract

Yb^3+^ and Y^3+^ double doped ZrO_2_ (8YSZ+4Yb_2_O_3_) samples were synthesized by a solid state reaction method. Moreover, 8YSZ+4Yb_2_O_3_-NaCl/KCl composites were also successfully produced at different temperatures. The 8YSZ+4Yb_2_O_3_, 8YSZ+4Yb_2_O_3_-NaCl/KCl (800 °C), and 8YSZ+4Yb_2_O_3_-NaCl/KCl (1000 °C) samples were characterized by x–ray diffraction (XRD) and scanning electron microscopy (SEM). The results showed that a dense composite electrolyte was formed at a low temperature of 800 °C. The maximum conductivities of 4.7 × 10^−2^ S·cm^−1^, 6.1 × 10^−1^ S·cm^−1^, and 3.8 × 10^−1^ S·cm^−1^ were achieved for the 8YSZ+4Yb_2_O_3_, 8YSZ+4Yb_2_O_3_-NaCl/KCl (800 °C), and 8YSZ+4Yb_2_O_3_-NaCl/KCl (1000 °C) samples at 700 °C, respectively. The logσ~log (*p*O_2_) plot result showed that the 8YSZ+4Yb_2_O_3_-NaCl/KCl (800 °C) composite electrolyte is a virtually pure ionic conductor. An excellent performance of the 8YSZ+4Yb_2_O_3_-NaCl/KCl (800 °C) composite was obtained with a maximum power density of 364 mW·cm^−2^ at 700 °C.

## 1. Introduction

Solid electrolytes for high temperature fuel cells have many advantages over liquid electrolytes such as high power density, good sealing, a broad test temperature range, etc. [1,2,3,4,5,6,7,8,9,10,11,12,13]. Electrolytes based on an oxide ion conducting divalent or trivalent cations stabilized zirconia have been widely studied [14,15,16,17]. In order to avoid deleterious phase transition, a stable tetragonal (cubic) structure of doped ZrO_2_ electrolytes can also be obtained. For instance, N.M. Rendtorff et al. synthesized the tetragonal structure of 3 mol % Y_2_O_3_ stabilized ZrO_2_ (3YSZ) through the mechanochemical activation technique [14]. However, solid oxide fuel cells (SOFCs) using cation stabilized ZrO_2_ as electrolytes have usually operated at high test temperature (800–1000 °C) as the conductivity of doped ZrO_2_ is significantly reduced for temperatures lower than 800 °C.

Composite electrolyte materials, which have high conductivities, are pivotal to the development of intermediate temperature fuel cells. In recent years, two-phase composite electrolytes consisting of doped BaCeO_3_, SrCeO_3_, CeO_2_, chloride, and carbonate have exhibited enhanced ionic conductivities and intermediate temperature fuel cell performance [18,19,20,21,22,23,24]. For example, Park et al. combined a perovskite-type BaCeO_3_ based electrolyte with a binary eutectic carbonate to obtain a high ionic conductivity of 0.176 S·cm^−1^ at 550 °C [18]. Fu et al. studied a gadolinium-doped ceria chloride composite electrolyte with a good power output density of 240 mW·cm^−2^ at 500 °C [24].

Many studies have shown that double cation doped ZrO_2_ could reduce the test temperature and improve its conductivity when compared to the single cation stabilized zirconia [25,26,27,28,29,30]. For instance, Liu et al. found that 1 wt % Al_2_O_3_ doped 8 mol % Y_2_O_3_ stabilized ZrO_2_ (8YSZ) reduced the sintering temperature of YSZ and improved the output of the fuel cell [26]. Wang et al. stabilized the structure phase when the substitution of Yb_2_O_3_ was over 2 mol % in the Yb_2_O_3_-Sc_2_O_3_-ZrO_2_ system. Furthermore, the radius of Yb^3+^ and Y^3+^ were both close to that of Zr^4+^ [27]. Therefore, we tried to fabricate a new composite electrolyte by using Yb^3+^ and Y^3+^ double doped ZrO_2_ together with a binary eutectic chloride.

In this paper, new Yb_2_O_3_ doped Zr_0.92_Y_0.08_O_2-α_(8YSZ)-NaCl/KCl composite electrolytes were prepared at different temperatures. The morphology and structure were characterized and the ionic conductivity and fuel cell were systematically evaluated.

## 2. Experimental

Yb^3+^ and Y^3+^ double doped ZrO_2_ was produced via a solid state reaction method. A total of 4 mol % Yb_2_O_3_ (99.9%) and 8YSZ (Xuancheng Jingrui New Material Co., Ltd., Xuancheng, China, sol-gel method, 50 nm) powders were fully mixed in ethanol under continuous stirring with an agate mortar and dried by an infrared lamp three times. Subsequently, the obtained powder was calcined at 1200 °C for 6 h to obtain the 8YSZ and 8YSZ+4Yb_2_O_3_ samples.

The binary eutectic chloride of KCl (0.202 g)–NaCl (0.158 g) (mole ratio = 1:1) was heated at 700 °C twice [31]. All of the used reagents were analytical grade (Sinopharm Chemical Reagent Co., Ltd., Shanghai, China). Then, 80 wt % of the 8YSZ+4Yb_2_O_3_ and 20 wt % of the KCl/NaCl powders were mixed and ground. The mixtures were sieved through 200 mesh and pressed into round disks under 200 MPa. Finally, the obtained disks were heated at 800 °C and 1000 °C for 2 h, respectively, to obtain 8YSZ+4Yb_2_O_3_-NaCl/KCl composite electrolytes.

The crystalline phases of the 8YSZ+4Yb_2_O_3_, 8YSZ+4Yb_2_O_3_-NaCl/KCl (800 °C), and 8YSZ+4Yb_2_O_3_-NaCl/KCl (1000 °C) samples were determined by X-ray diffraction (XRD, X’pert Pro MPD, Holland’s company, Amsterdam, The Netherlands). The morphology of the sintered pellets was observed by using a scanning electron microscope (SEM, S-4700, Hitachi, Tokyo, Japan).

All samples were ground and pressed into thin slices (thickness = 1.0–1.2 mm) and 80% silver-20% palladium paste (areas = 0.5 cm^2^) was used with silver wires as the electrodes. Electrochemical impedance spectroscopy (EIS) techniques were used to obtain the conductivity of the 8YSZ+4Yb_2_O_3_, 8YSZ+4Yb_2_O_3_-NaCl/KCl (800 °C), and 8YSZ+4Yb_2_O_3_-NaCl/KCl (1000 °C). The ac amplitude was 20 mV in a three-electrode system over the frequency range from 1 Hz to 1 MHz. The conductivity can be calculated from: σ = $\frac{L}{R\cdot S}$, where σ is conductivity, *L* is thickness, *R* is resistance, and *S* is the surface area of the electrolyte pellet [32,33]. The effects of different synthetic temperature, operating temperature, and oxygen partial pressure on the electrical conductivities were determined with an electrochemical analyzer (CHI660E, Chen Hua company, Shanghai, China) at 400–700 °C [34,35]. Oxygen concentration discharge cell: air, Pd-Ag|8YSZ+4Yb_2_O_3_-NaCl/KCl (800 °C)|Pd-Ag, O_2_ at 700 °C and H_2_/O_2_ fuel cells using 8YSZ+4Yb_2_O_3_ and 8YSZ+4Yb_2_O_3_-NaCl/KCl (800 °C) as electrolytes (thickness = 1.1 mm) were constructed. The fuel cell was tested by using the linear scanning of current and voltage method within the CHI660E electrochemical analyzer [33].

## 3. Results and Discussion

**Figure 1:**
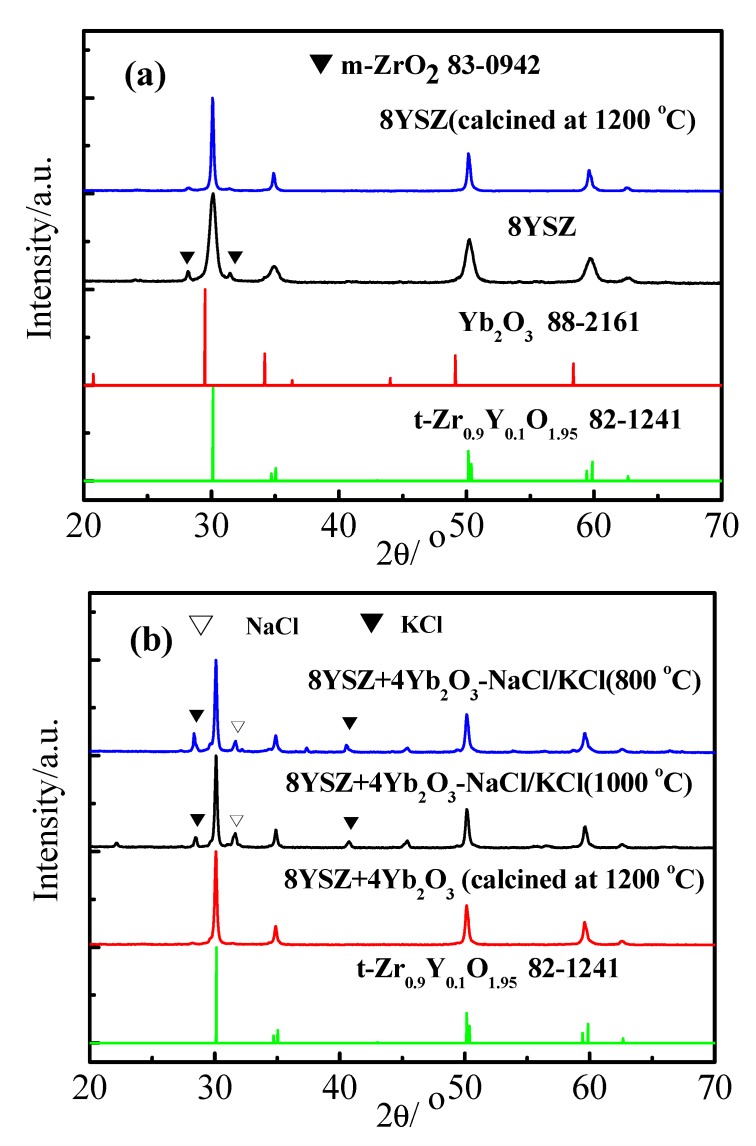
(**a**) XRD patterns of the 8YSZ and 8YSZ (1200 °C) samples. (**b**) XRD patterns of the 8YSZ+4Yb_2_O_3_, 8YSZ+4Yb_2_O_3_-NaCl/KCl (800 °C), and 8YSZ+4Yb_2_O_3_-NaCl/KCl (1000 °C) samples.

Figure 1 presents the X-ray diffraction (XRD) patterns of the 8YSZ, 8YSZ (1200 °C), 8YSZ+4Yb_2_O_3_ (1200 °C), 8YSZ+4Yb_2_O_3_-NaCl/KCl (800 °C), and 8YSZ+4Yb_2_O_3_-NaCl/KCl (1000 °C) samples together with the standard diffraction patterns of Yb_2_O_3_ (JCPDS 88-2161) and t-Zr_0.9_Y_0.1_O_1.95_ (JCPDS 82-1241). The 8YSZ (Xuancheng Jingrui New Material Co., Ltd., sol-gel method, 50 nm) powder possessed coexisting tetragonal and monoclinic phases, where the tetragonal was the major phase, as shown in Figure 1a. When the synthesis temperature reached 1200 °C, it was observed that the 8YSZ and 8YSZ+4Yb_2_O_3_ showed an entire t-ZrO_2_ phase. Yb^3+^ really formed a solid solution with YSZ and there was no trace of Yb_2_O_3_. Additionally, NaCl and KCl diffraction peaks also existed in the 8YSZ+4Yb_2_O_3_-NaCl/KCl (800 °C) and 8YSZ+4Yb_2_O_3_-NaCl/KCl (1000 °C) samples, that is to say that the binary eutectic chloride did not react with 8YSZ+4Yb_2_O_3_. This was in good agreement with the studies of ceria-carbonates or doped SrCeO_3_-NaCl-KCl composite electrolytes [36,37].

**Figure 2:**
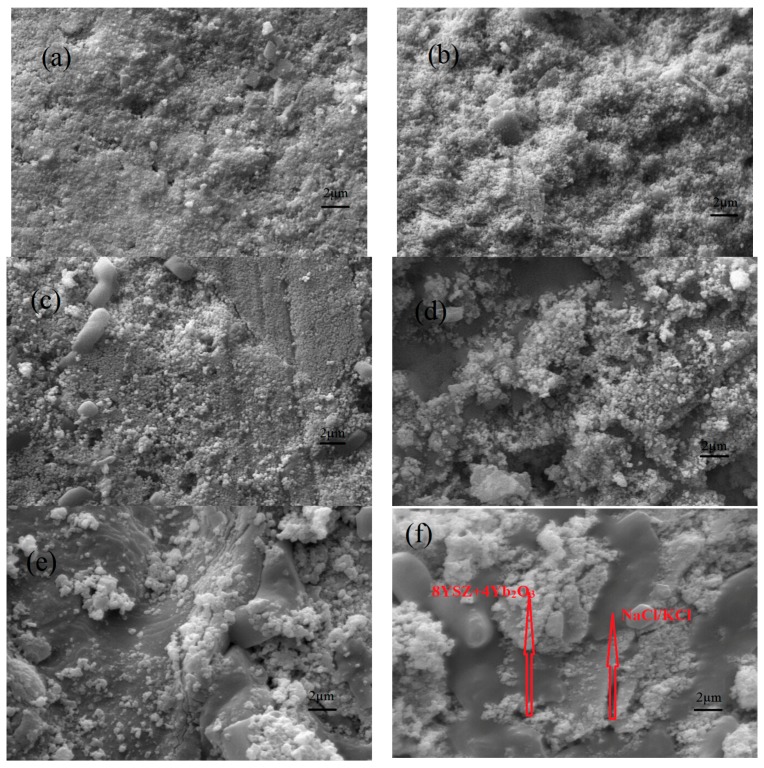
Surface (**a**,**c**,**e**) and cross-sectional (**b**,**d**,**f**) SEM images of the 8YSZ+4Yb_2_O_3_ (**a**,**b**), 8YSZ+4Yb_2_O_3_-NaCl/KCl (1000 °C) (**c**,**d**), and 8YSZ+4Yb_2_O_3_-NaCl/KCl (800 °C) (**e**,**f**) pellets.

The surface (a,c,e) and cross-sectional (b,d,f) morphologies of the 8YSZ+4Yb_2_O_3_, 8YSZ+4Yb_2_O_3_-NaCl/KCl (800 °C) ,and 8YSZ+4Yb_2_O_3_-NaCl/KCl (1000 °C) pellets, as seen using SEM, are exhibited in Figure 2. No pores or cracks on the surface (e) and cross-sectional (f) photos of the 8YSZ+4Yb_2_O_3_-NaCl/KCl (800 °C) were seen along the entire experiment, which meant that the Yb^3+^ and Y^3+^ double doped ZrO_2_ and the binary eutectic chloride sintered uniformly. The 8YSZ+4Yb_2_O_3_ and NaCl/KCl are indicated by arrows in Figure 2f. However, as can be observed from Figure 2c,d, a few scattered small pores were found in the 8YSZ+4Yb_2_O_3_-NaCl/KCl (1000 °C) pellet, which may influence the electrical properties of the sample. The sintering performance of the 8YSZ+4Yb_2_O_3_-NaCl/KCl pellet increased with the increase in temperature, nevertheless, the vapor pressure of the molten inorganic salts rapidly increased at the same time. This was consistent with the ceria-carbonates or doped SrCeO_3_-chloride composite electrolytes prepared under similar heat treatments [36,37,38,39]. Therefore, the most suitable synthetic temperature was 800 °C.

**Figure 3:**
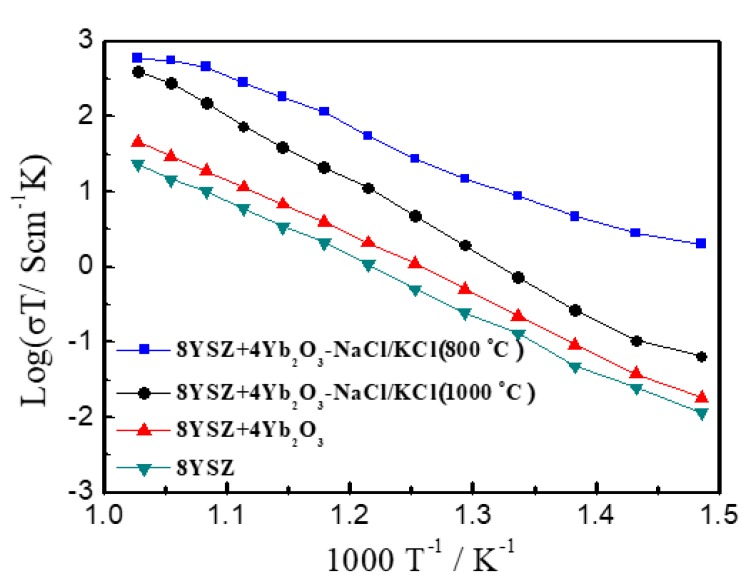
The log (σT)~1000 T^−1^ plots of the 8YSZ, 8YSZ+4Yb_2_O_3_, 8YSZ+4Yb_2_O_3_-NaCl/KCl (800 °C), and 8YSZ+4Yb_2_O_3_-NaCl/KCl (1000 °C) pellets in air at 400–700 °C.

The plots of log (σT) versus 1000 T^−1^ of the 8YSZ, 8YSZ+4Yb_2_O_3_, 8YSZ+4Yb_2_O_3_-NaCl/KCl (800 °C), and 8YSZ+4Yb_2_O_3_-NaCl/KCl (1000 °C) pellets in air at 400–700 °C are given in Figure 3. The maximum conductivities achieved for 8YSZ+4Yb_2_O_3_, 8YSZ+4Yb_2_O_3_-NaCl/KCl (800 °C), and 8YSZ+4Yb_2_O_3_-NaCl/KCl (1000 °C) were 4.7 × 10^−2^ S·cm^−1^, 6.1 × 10^−1^ S·cm^−1^, and 3.8 × 10^−1^ S·cm^−1^ at 700 °C, respectively. Wang et al. [27] demonstrated that the conductivities of Yb^3+^ and Sc^3+^ double doped ZrO_2_ could maintain 1.0 × 10^−2^ S·cm^−1^ at 700 °C, which is the threshold value for application as an electrolyte. The experimental result of Bohnke et al. [28] showed that the conductivity of the (Sc_2_O_3_)_0.07_-(Fe_2_O_3_)_0.03_-(ZrO_2_)_0.90_ was lower than 1.0 × 10^−2^ S·cm^−1^ at 700 °C. Our result was equivalent to the former. The conductivities of electrolytes have been generally found to be higher in a wet atmosphere in comparison with a dry atmosphere [33]. Furthermore, 8YSZ+4Yb_2_O_3_ exhibited a highest electrical conductivity of 4.7 × 10^−2^ S·cm^−1^ when compared to the 8YSZ of 2.3 × 10^−2^ S·cm^−1^ at 700 °C. The measured conductivities of the composite electrolytes were much higher than those of 8YSZ+4Yb_2_O_3_ and in our previous study of SrCe_0.9_Sm_0.1_O_3-α_-NaCl-KCl (1.43 × 10^−1^ S·cm^−1^). This revealed that the introduction of the binary eutectic chloride allows for ionic charge carriers to move quickly and freely through it, which is beneficial for the long-range transfer ability of ions [36,40]. In Figure 3, with the increase in sintering temperature, the conductivities of the 8YSZ+4Yb_2_O_3_-NaCl/KCl samples decreased. The conductivity of 8YSZ+4Yb_2_O_3_-NaCl/KCl (1000 °C) was lower than that of the sample sintered at 800 °C, especially at a low temperature range. This might be associated with the breaking of the long-range transfer of ionic charge carriers to a certain extent after heating at 1000 °C, according to the result of Figure 2.

**Figure 4:**
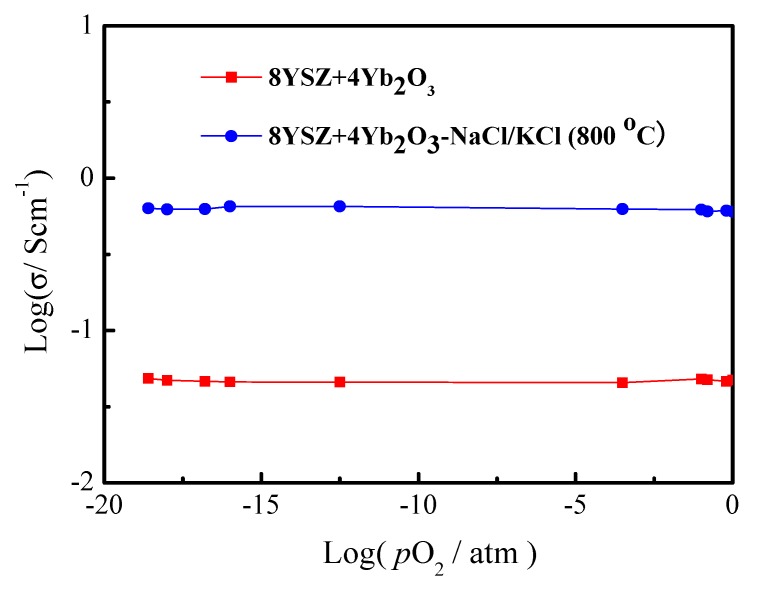
The conductivities of the 8YSZ+4Yb_2_O_3_ and 8YSZ+4Yb_2_O_3_-NaCl/KCl (800 °C) as a function of *p*O_2_ at 700 °C.

To reveal the ionic conduction of the 8YSZ+4Yb_2_O_3_ and 8YSZ+4Yb_2_O_3_-NaCl/KCl (800 °C) samples, the variation of the conductivities with the partial pressures of oxygen in the range of *p*O_2_ = 10^−20^~1 atm were evaluated. It can be seen from Figure 4 that the conductivities were almost independent of *p*O_2_ at 700 °C, implying that the 8YSZ+4Yb_2_O_3_-NaCl/KCl (800 °C) composite electrolyte is a virtually pure ionic conductor, which agrees with previous reports [20,37,38]. The result illustrates that the conductivities of the two electrolytes in Figure 3 are purely ionic.

**Figure 5:**
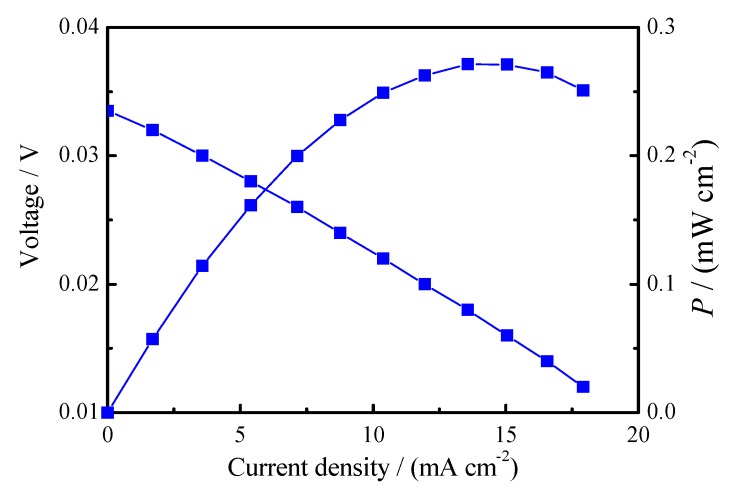
The oxygen concentration discharge cell: air, Pd-Ag|8YSZ+4Yb_2_O_3_-NaCl/KCl (800 °C)|Pd-Ag, O_2_ at 700 °C.

To explore the oxide ionic conduction of the 8YSZ+4Yb_2_O_3_-NaCl/KCl (800 °C) composite under an oxygen-containing atmosphere, an oxygen concentration discharge cell was fabricated and tested at 700 °C, as illustrated in Figure 5. The calculated electromotive forces (EMF_cal_) of the oxygen concentration discharge cell can be obtained from EMF_cal_ = $\frac{RT}{4F}$ *t*_O_ ln[*p*O_2(A)_/*p*O_2(B)_] when *t*_O_ = 1. It was obvious that the measured open circuit voltage exhibited a value of 33 mV, which was consistent with the calculated EMF (32.7 mV). In addition, the 8YSZ+4Yb_2_O_3_-NaCl/KCl (800 °C) composite was believed to be an oxide ionic conductor under an oxygen-containing atmosphere due to the exhibited stable discharge curve [41,42].

**Figure 6:**
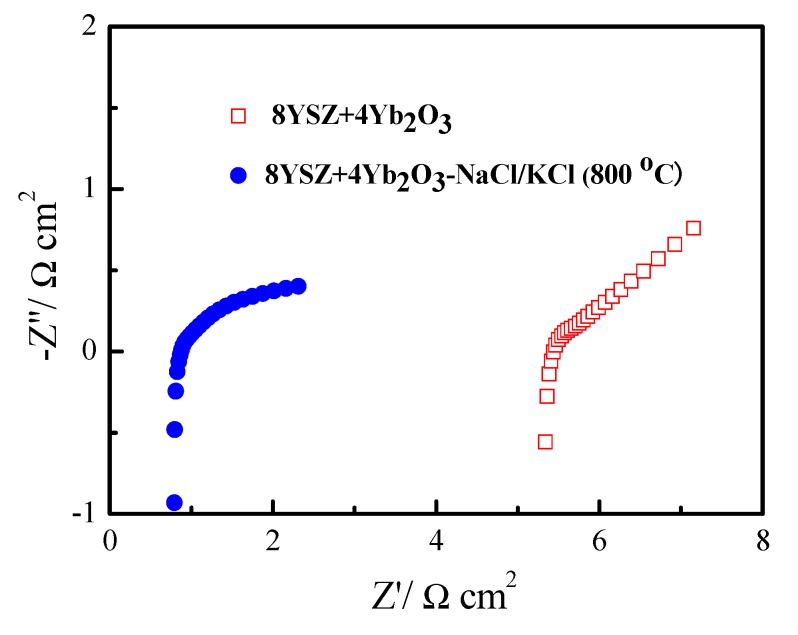
Typical impedance spectra of the 8YSZ+4Yb_2_O_3_ and 8YSZ+4Yb_2_O_3_-NaCl/KCl (800 °C) samples measured at 700 °C under open-circuit conditions.

Figure 6 is the typical impedance spectra of the 8YSZ+4Yb_2_O_3_ and 8YSZ+4Yb_2_O_3_-NaCl/KCl (800 °C) measured at 700 °C under open-circuit conditions. The spectra gave an incomplete semicircle at intermediate to high frequencies, and an arc at low frequency. The high frequency related to the ohmic resistance (*R*_o_) and the low frequency can be attributed to the resistance between the electrode and the electrolyte [43]. The interval between the high and low frequencies corresponded to interfacial polarization resistance (*R*_p_) where the *R*_p_ and *R*_o_ of 8YSZ+4Yb_2_O_3_ were 0.41 Ω·cm^2^ and 5.45 Ω·cm^2^, while those of 8YSZ+4Yb_2_O_3_-NaCl/KCl (800 °C) were 0.16 Ω·cm^2^ and 0.87 Ω·cm^2^ at 700 °C, correspondingly.

**Figure 7:**
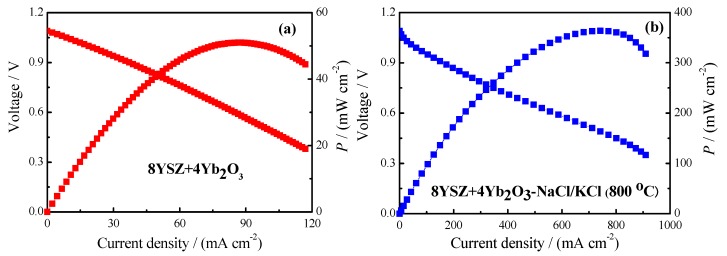
*I*-*V* and *I*-*P* curves based on 8YSZ+4Yb_2_O_3_ and 8YSZ+4Yb_2_O_3_-NaCl/KCl (800 °C) at 700 °C.

The single cells based on 8YSZ+4Yb_2_O_3_ and 8YSZ+4Yb_2_O_3_-NaCl/KCl (800 °C) were operated at 700 °C, hydrogen and oxygen were supplied at the anode side and cathode side, respectively, and the *I-V* and power density curves are displayed in Figure 7. The following two reactions occur at the cathode and anode compartments: cathode reaction: 2H^+^ + O_2_ + 4e^−^ = H_2_O + O^2−^ and anode reaction: 2H_2_ + O^2−^ = 2H^+^ + H_2_O + 4e^−^ [44]. As can be observed from Figure 7, the open circuit voltages were as high as 1.09 V, which confirmed that the samples possessed high densities. This can be attributed to the fact that NaCl–KCl eutectic melt fills the pores inside the composite electrolyte, leading to the increase in the density of the composite electrolyte at 700 °C. In this state, the 8YSZ+4Yb_2_O_3_ electrolyte and NaCl/KCl were between the continuous and discontinuous phases. The conductivities can be ascribed to the mobility of various species (Na^+^, K^+^, H^+^, Cl^−^ and O^2^^−^). And protons vacancies are the predominated defects under wet conditions, especially in a hydrogen containing atmosphere. Therefore, the fuel cell based on 8YSZ+4Yb_2_O_3_-NaCl/KCl (800 °C) exhibited good cell performance and gave a power output density of 364 mW·cm^−2^ at 700 °C. The result was much better than the best performance of 8YSZ+4Yb_2_O_3_ and that ever reported for intermediate temperature SOFCs based on SrCe_0.9_Eu_0.1_O_3-α_-NaCl-KCl of 207 mW·cm^−2^ [37] at 700 °C. However, the durabilities of the electrolytes were not been tested, and will be done in future work.

## 4. Conclusions

In this study, Yb^3+^ and Y^3+^ double doped ZrO_2_ and its composite electrolytes were successfully fabricated by a solid state reaction method. The result of the logσ~log (*p*O_2_) plot indicated that the 8YSZ+4Yb_2_O_3_-NaCl/KCl (800 °C) composite electrolyte was a virtually pure ionic conductor. Furthermore, the oxygen concentration discharge cell illustrated that the 8YSZ+4Yb_2_O_3_-NaCl/KCl (800 °C) composite was an oxide ionic conductor under an oxygen-containing atmosphere. The *R*_p_ and *R*_o_ were 0.41 Ω·cm^2^ and 5.45 Ω·cm^2^ for 8YSZ+4Yb_2_O_3_, and 0.16 Ω·cm^2^, and 0.87 Ω·cm^2^ for 8YSZ+4Yb_2_O_3_-NaCl/KCl (800 °C) under open-circuit conditions at 700 °C correspondingly. Finally, an excellent performance of 8YSZ+4Yb_2_O_3_-NaCl/KCl (800 °C) was obtained with a maximum power density of 364 mW·cm^−2^ at 700 °C.

## References

[1] Hibino T., Kobayashi K., Lv P., Nagao M., Teranishi S., Mori T. (2017). An intermediate- temperature biomass fuel cell using wood sawdust and pulp directly as fuel. J. Electrochem. Soc..

[2] Sadeghifar H. (2017). In-plane and through-plane electrical conductivities and contact resistances of a Mercedes-Benz catalyst-coated membrane, gas diffusion and micro-porous layers and a Ballard graphite bipolar plate: Impact of humidity, compressive load and polytetrafluoroethylene. Energy Convers. Manag..

[3] Irshad M., Siraj K., Raza R., Ali A., Tiwari P., Zhu B., Asia R., Ali A., Ullah M.K., Usman A. (2016). A brief description of high temperature solid oxide fuel cell’s operation, materials, design, fabrication technologies and performance. Appl. Sci..

[4] Sadeghifar H., Bahrami M., Djilali N. (2013). A statistically-based thermal conductivity model for fuel cell Gas Diffusion Layers. J. Power Sources.

[5] Xia C., Qiao Z., Feng C., Kim J., Wang B., Zhu B. (2018). Study on zinc oxide-based electrolytes in low-temperature solid oxide fuel cells. Materials.

[6] Fang X., Zhu J., Lin Z. (2018). Effects of electrode composition and thickness on the mechanical performance of a solid oxide fuel cell. Energies.

[7] Sadeghifar H., Djilali N., Bahrami M. (2014). Effect of Polytetrafluoroethylene (PTFE) and micro porous layer (MPL) on thermal conductivity of fuel cell gas diffusion layers: Modeling and experiments. J. Power Sources.

[8] Sadeghifar H., Djilali N., Bahrami M. (2014). A new model for thermal contact resistance between fuel cell gas diffusion layers and bipolar plates. J. Power Sources.

[9] Bernuy-Lopez C., Rioja-Monllor L., Nakamura T., Ricote S., O’Hayre R., Amezawa K., Einarsrud M., Grande T. (2018). Effect of cation ordering on the performance and chemical stability of layered double perovskite cathodes. Materials.

[10] Vilela C., Martins A.P.C., Sousa N., Silvestre A.J.D., Figueiredo F.M.L., Freire C.S.R. (2018). Poly(bis [2-(methacryloyloxy) ethyl] phosphate)/bacterial cellulose nanocomposites: Preparation, characterization and application as polymer electrolyte membranes. Appl. Sci..

[11] Hibino T., Kobayashi K., Nagao M., Teranishi S. (2017). Hydrogen production by direct lignin electrolysis at intermediate temperatures. ChemElectroChem.

[12] Sadeghifar H., Djilali N., Bahrami M. (2015). Thermal conductivity of a graphite bipolar plate (BPP) and its thermal contact resistance with fuel cell gas diffusion layers: Effect of compression, PTFE, micro porous layer (MPL), BPP out-of-flatness and cyclic load. J. Power Sources.

[13] Luo J., Jensen A.H., Brooks N.R., Sniekers J., Knipper M., Aili D., Li Q., Vanroy B., Wübbenhorst M., Yan F. (2015). 1,2,4-Triazolium perfluorobutanesulfonate as an archetypal pure protic organic ionic plastic crystal electrolyte for all-solid-state fuel cells. Energy Environ. Sci..

[14] Rendtorff N.M., Suarez G., Aglietti E.F., Rivas P.C., Martinez J.A. (2013). Phase evolution in the mechanochemical synthesis of stabilized nanocrystalline (ZrO_2_)_0.97_(Y_2_O_3_)_0.03_ solid solution by PAC technique. Ceram. Int..

[15] Liu X.Y., Xu Z.H., Liang G.Y. (2017). Comparative study of the sintering behaviors between YSZ and LZ/YSZ composite. Mater. Lett..

[16] Dankeaw A., Poungchan G., Panapoy M., Ksapabutr B. (2017). In-situ one-step method for fabricating three-dimensional grass-like carbon-doped ZrO_2_ films for room temperature alcohol and acetone sensors. Sens. Actuators B Chem..

[17] Mamana N., Díaz-Parralejo A., Ortiz A.L., Sánchez-Bajo F., Caruso R. (2014). Influence of the synthesis process on the features of Y_2_O_3_-stabilized ZrO_2_ powders obtained by the sol-gel method. Ceram. Int..

[18] Park K.-Y., Lee T.-H., Kim J.-T., Lee N., Seo Y., Song S.-J., Park J.-Y. (2014). Highly conductive barium zirconate-based carbonate composite electrolytes for intermediate temperature-protonic ceramic fuel cells. J. Alloys Compd..

[19] Slim C., Baklouti L., Cassir M., Ringuedé A. (2014). Structural and electrochemical performance of gadolinia-doped ceria mixed with alkali chlorides (LiCl-KCl) for Intermediate Temperature-Hybrid Fuel Cell applications. Electrochim. Acta.

[20] Zhang W., Yuan M., Wang H., Liu J. (2016). High-performance intermediate temperature fuel cells of new SrCe_0.9_Yb_0.1_O_3-__α_-inorganic salt composite electrolytes. J. Alloys Compd..

[21] Ojha A.K., Ponnilavan V., Kannan S. (2017). Structural, morphological and mechanical investigations of in situ synthesized c-CeO_2_/α-Al_2_O_3_ composites. Ceram. Int..

[22] Martins N.C.T., Rajesh S., Marques F.M.B. (2015). Synthesis and electrochemical assessment of Ce_0.5_Yb_0.5_O_1.75_ ceramics and derived composite electrolytes. Mater. Res. Bull..

[23] Kim J.-T., Lee T.-H., Park K.-Y., Seo Y., Kim K.B., Song S.-J., Park B., Park J.-Y. (2015). Electrochemical properties of dual phase neodymium-doped ceria alkali carbonate composite electrolytes in intermediate temperature. J. Power Sources.

[24] Fu Q.X., Zhang W., Peng R.R., Peng D.K., Meng G.Y., Zhu B. (2002). Doped ceria–chloride composite electrolyte for intermediate temperature ceramic membrane fuel cells. Mater. Lett..

[25] Kravchyk K.V., Bohnke O., Gunes V., Belous A.G., Pashkova E.V., Lannic J.L., Gouttefangeas F. (2012). Ionic and electronic conductivity of 3 mol% Fe_2_O_3_-substituted cubic Y-stabilized ZrO_2_. Solid State Ion..

[26] Lei L., Bai Y., Liu J. (2014). Ni-based anode-supported Al_2_O_3_-doped-Y_2_O_3_-stabilized ZrO_2_ thin electrolyte solid oxide fuel cells with Y_2_O_3_-stabilized ZrO_2_ buffer layer. J. Power Sources.

[27] Yuan F., Wang J.X., Miao H., Guo C., Wang W. (2013). Investigation of the crystal structure and ionic conductivity in the ternary system (Yb_2_O_3_)_x_–(Sc_2_O_3_)_(0.11−x)_–(ZrO_2_)_0.89_ (x = 0–0.11). J. Alloys Compd..

[28] Bohnke O., Gunes V., Kravchyk K.V., Belous A.G., Yanchevskii O.Z., V’Yunov O.I. (2014). Ionic and electronic conductivity of 3 mol% Fe_2_O_3_-substituted cubic yttria-stabilized ZrO_2_ (YSZ) and scandia-stabilized ZrO_2_ (ScSZ). Solid State Ion..

[29] Chen Y., Orlovskaya N., Payzant E.A., Graule T., Kuebler J. (2015). A search for temperature induced time-dependent structural transitions in 10 mol% Sc_2_O_3_–1 mol% CeO_2_–ZrO_2_ and 8mol% Y_2_O_3_–ZrO_2_ electrolyte ceramics. J. Eur. Ceram. Soc..

[30] Zeeshan N., Rafiuddin (2018). Solid electrolytes based on {1 − (x + y)}ZrO_2_-(x)MgO-(y)CaO ternary system: Preparation, characterization, ionic conductivity, and dielectric properties. J. Adv. Res..

[31] Liu X., Fechler N., Antonietti M. (2013). Salt melt synthesis of ceramics, semiconductors and carbon nanostructures. Chem. Soc. Rev..

[32] Sadeghifar H., Djilali N., Bahrami M. (2014). Counter-intuitive reduction of thermal contact resistance with porosity: A case study of polymer electrolyte membrane fuel cells. Int. J. Hydrog. Energy.

[33] Shi R., Chen W., Hu W., Liu J., Wang H. (2018). SrCe_0.9_Sm_0.1_O_3-α_ Compounded with NaCl-KCl as a Composite Electrolyte for Intermediate Temperature Fuel Cell. Materials.

[34] Sadeghifar H. (2018). An optimized microstructure to minimizing in-plane and through-plane pressure drops of fibrous materials: Counter-intuitive reduction of gas diffusion layer permeability with porosity. J. Power Sources.

[35] Sadeghifar H. (2016). In-plane and through-plane local and average Nusselt numbers in fibrous porous materials with different fiber layer temperatures: Gas diffusion layers for fuel cells. J. Power Sources.

[36] Zhu B., Li S., Mellander B.E. (2008). The oretical approach on ceria-based two-phase electrolytes for low temperature (300–600 °C) solid oxide fuel cells. Electrochem. Commun..

[37] Shi R., Liu J., Wang H., Wu F., Miao H., Cui Y. (2017). Low temperature synthesis of SrCe_0.9_Eu_0.1_O_3-α_ by sol-gel method and SrCe_0.9_Eu_0.1_O_3-α_-NaCl-KCl composite electrolyte for intermediate temperature fuel cells. Int. J. Electrochem. Sci..

[38] Sun L., Miao H., Wang H. (2017). Novel SrCe_1-x_Yb_x_O_3-α_-(Na/K)Cl composite electrolytes for intermediate temperature solid oxide fuel cells. Solid State Ion..

[39] Afzal M., Raza R., Du S., Lima R.B., Zhu B. (2015). Synthesis of Ba_0.3_Ca_0.7_Co_0.8_Fe_0.2_O_3-α_ composite material as novel catalytic cathode for ceria-carbonate electrolyte fuel cells. Electrochim. Acta.

[40] Sadeghifar H. (2016). Reconstruction and analysis of fuel cell gas diffusion layers using fiber spacing rather than pore size data: Questioned validity of widely-used porosity-based thermal conductivity models. J. Power Sources.

[41] Liu J., Du R., Shi R., Wang H. (2018). Facile Synthesis and Enhanced Intermediate Temperature Electrical Properties of Novel Sn_0.9_Mg_0.1_P_2_O_7_/KSn_2_(PO_4_)_3_ Composite Electrolyte. Int. J. Electrochem. Sci..

[42] Ma G., Shimura T., Iwahara H. (1999). Simultaneous doping with La^3+^ and Y^3+^ for Ba^2+^- and Ce^4+^-sites in BaCeO_3_ and the ionic conduction. Solid State Ion..

[43] Baek S.-S., Park K.-Y., Lee T.-H., Lee N., Seo Y., Song S.-J., Park J.-Y. (2014). PdO-doped BaZr_0.8_Y_0.2_O_3−δ_ electrolyte for intermediate-temperature protonic ceramic fuel cells. Acta Mater..

[44] Guo Y., Liu B., Yang Q., Chen C., Wang W., Ma G. (2009). Preparation via microemulsion method and proton conduction at intermediate-temperature of BaCe_1-x_Y_x_O_3-α_. Electrochem. Commun..

